# 3D LiDAR Point Cloud Registration Based on IMU Preintegration in Coal Mine Roadways

**DOI:** 10.3390/s23073473

**Published:** 2023-03-26

**Authors:** Lin Yang, Hongwei Ma, Zhen Nie, Heng Zhang, Zhongyang Wang, Chuanwei Wang

**Affiliations:** 1 School of Mechanical Engineering, Xi’an University of Science and Technology, Xi’an 710054, China; 2 Shaanxi Key Laboratory of Mine Electromechanical Equipment Intelligent Monitoring, Xi’an 710054, China

**Keywords:** point cloud registration, coal mine roadway, distortion correction, ground segmentation, feature extraction

## Abstract

Point cloud registration is the basis of real-time environment perception for robots using 3D LiDAR and is also the key to robust simultaneous localization and mapping (SLAM) for robots. Because LiDAR point clouds are characterized by local sparseness and motion distortion, the point cloud features of coal mine roadway environments show a weak texture and degradation. Therefore, for these environments, the traditional point cloud registration method to register directly will lead to problems, such as a decline in registration accuracy, z-axis drift, and map ghosting. To solve the above problems, we propose a point cloud registration method based on IMU preintegration with the sensor characteristics of LiDAR and IMU. The system framework of this method mainly consists of four modules: IMU preintegration, point cloud preprocessing, point cloud frame matching and point cloud registration. First, IMU sensor data are introduced, and IMU linear interpolation is used to correct the motion distortion in LiDAR scanning, and the IMU preintegration error function is constructed. Second, the point cloud segmentation is performed using the ground segmentation method of RANSAC to provide additional ground constraints for the z-axis displacement and to remove the unstable flawed points from the point cloud. On this basis, the LiDAR point cloud registration error function is constructed by extracting the feature corner points and feature plane points. Finally, the Gaussian Newton solution is used to optimize the constraint relationship between the LiDAR odometry frames to minimize the error function, complete the LiDAR point cloud registration and better estimate the position and pose of the mobile robot. The experimental results show that compared with the traditional point cloud registration method, the proposed method has a higher point cloud registration accuracy, success rate and computational efficiency. The LiDAR odometry constructed using this method can better reflect the authenticity of the robot trajectory and has higher trajectory accuracy and smaller absolute position and pose error.

## 1. Introduction

Due to the characteristics of high dust, low illumination and weak texture of coal mine roadways, it is difficult for visual sensors to extract stable feature points. In contrast, the biggest advantage of 3D LiDAR is that it is not affected by light in the coal mine roadway environment and can provide long-distance, centimetre-level measurement accuracy. Therefore, as the most important sensor in robot sensing systems, LiDAR has been widely used in the functions of coal mine robots, such as simultaneous localization and mapping (SLAM) [1], precise positioning [2,3], map generation [4], and target detection [5]. However, the use of LiDAR sensors in coal mines currently is associated with problems, such as measurement noise, range limitation, environmental occlusion, and feature degradation in the scanning process. As a result, the collected LiDAR point cloud has motion distortion, feature degradation, 3D information loss and other problems, which often makes the point cloud registration fail in the corner of feature degradation or in the scene of rapid conversion. Because IMU sensors can provide high-precision state estimation in a short time, it is not subject to environmental and structural changes. Therefore, in order to make up for the defects of laser radar sensors, the method of LiDAR and IMU multi-sensor fusion is usually adopted to improve the robustness of mobile robots to adapt to the complex environment of coal mine roadways.

The most important step of LiDAR scanning is point cloud registration. The existing work based on LiDAR SLAM usually describes the point cloud registration problem as two modules: scan-to-scan matching and scan-to-map refinement. These two modules are solved by iterative calculation, and the calculation cost is high. The iterative closest point (ICP) [6] is the most classical method to estimate the transformation relationship between two LiDAR scans, in which the two scans are aligned iteratively by minimizing the point cloud distance. However, the optimization process involves many point-to-point matches and strongly relies on the nearest neighbour search to associate the nearest point, which has low computational efficiency. Unlike the ICP algorithm, the normal distribution transformation (NDT) algorithm [7] is a distribution-based method and does not rely on accurately calculating the nearest neighbour search of a single point. This method optimizes the conversion by measuring the normal distribution of each cell in the probability of a point. However, although this method improves the registration efficiency, it loses registration accuracy. To reduce computational complexity and obtain accurate registration results, feature-based registration methods have been gradually proposed. A typical example is LiDAR odometry and mapping (LOAM) [8], which extracts edge and plane features and realizes low drift and real-time state estimation and map construction by performing point-to-line and point-to-plane matching. Because these features can robustly find the corresponding relationship between point clouds, feature-based methods are usually robust to initial attitude errors. However, feature-based methods only use a limited number of features, so they are less accurate than point-based methods. In recent years, some work related to deep learning has also been used for point cloud registration research. Although the convolutional neural network (CNN) method has shown good performance in public datasets, the robustness of mobile robots in different environments is poor. Many methods only focus on the point cloud registration stage but ignore the importance of feature extraction [9,10].

The purpose of point cloud registration is to align the LiDAR point cloud data of different frames into the specified coordinate system through rigid transformations, such as rotation and translation, and finally solve the absolute pose change relationship of the LiDAR coordinate system. Although existing LiDAR point cloud scanning registration research has achieved good performance in 3D reconstruction, some problems persist in the practical application of mobile robots. For example, when the robot collects the LiDAR point cloud data in the process of motion, asynchronous LiDAR measurements are easily generated due to the rotation characteristics of the rotating LiDAR and the sequential manner of generating the measurement results. As a result, the point cloud motion is distorted, which reduces the accuracy of the laser odometer or the overall performance of the SLAM. When the ground mobile robot only uses LiDAR to match the point cloud, the relative transformation of 3D LiDAR data is mainly related to the ground direction because the ground mobile robot can only move on the ground plane along the horizontal direction. Therefore, the transformation in the x-axis, y-axis and yaw angle directions is large, while the transformation in the z-axis, roll angle and pitch angle directions is small [11]. As a result, significant deviations are generated in the fused point cloud scene on the z-axis, causing ghost or deformation phenomena in the constructed point cloud scene that decrease the accuracy of point cloud registration. When the robot works in the coal mine roadway environment, if the point cloud of the current frame and the target frame is directly registered with the point cloud due to the local sparsity of the LiDAR point cloud data and the degradation of the point cloud characteristics, the accuracy of the point cloud registration will be reduced, and the positioning and mapping performance of the robot will be seriously affected.

To improve the performance of ground mobile robots in the special environment of coal mine roadways and facilitate high-performance point cloud registration, we propose a point cloud registration method with ground segmentation and IMU preintegration based on the motion characteristics of the ground mobile robot. The goal is to provide a real-time, efficient, accurate and robust LiDAR point cloud registration scheme for coal mine roadway mobile robots. Because most points in the 3D point cloud on the ground mobile robot are from the ground in the environment, a ground segmentation point cloud preprocessing method is used to help roughly extract the plane features of the robot’s surrounding environment and improve the efficiency of point cloud feature extraction. We have designed a new framework combining an IMU preintegration module, a point cloud preprocessing module, a point cloud frame matching module, and a point cloud registration module so that robot LiDAR scanning has better adaptability and is especially suitable for the special environment of coal mine roadways. However, the mobile robot has limitations for the coal mine roadway environment. How to ensure the effective balance between the point cloud registration accuracy, registration success rate, and computational complexity, while improving the robustness of point cloud registration, is still a major problem. Therefore, research focused on this topic is of great theoretical research value and practical application significance.

In summary, our contributions mainly include the following three aspects:

1. We propose a point cloud registration method based on IMU preintegration. The system framework of this method mainly consists of four modules: IMU preintegration, point cloud preprocessing, point cloud frame matching and point cloud registration. The results show that our point cloud registration method has a higher accuracy, success rate and computational efficiency.
2. The problem of point cloud distortion in LiDAR scanning is solved by introducing IMU sensor data and using the IMU linear interpolation correction method, which improves the quality of the original point cloud; the ground segmentation method using RANSAC to provide additional ground constraints for z-axis displacement; the stability of point cloud registration is improved by eliminating the unstable flawed points; the robustness of point cloud registration is improved by extracting the feature corner points and feature plane points from the point cloud.
3. This method uses Gauss-Newton to solve the constraint relationship between IMU preintegration error and LiDAR registration error, so as to minimize the error function, complete the optimal registration of the LiDAR point cloud, and better estimate the pose of the mobile robot. Aiming at the special environment of point cloud feature degradation in coal mine roadways, compared with the method based on GICP, the LiDAR odometry constructed by using our point cloud registration method has higher track accuracy and can better reflect the authenticity of the track.

The remainder of this paper is organized as follows. In Section 2, we discuss the relevant work of point cloud registration research. We outline the complete system framework and a detailed system overview and describe in detail the specific approach and methodology of each module in Section 3. On this basis, we analyse the relevant results and discussed them in Section 4, followed by conclusions in Section 5.

## 2. Related Works

3D LiDAR point cloud registration is the core of LiDAR odometry and LiDAR-SLAM. It is the key basis for simultaneous localization and mapping construction using LiDAR sensors. It is the most common method for LiDAR-SLAM to achieve data association. Existing 3D LiDAR point cloud registration methods are mainly divided into three categories: point-based methods, distribution-based methods and feature-based methods.

### 2.1. Point-Based Method

The iterative closest point (ICP) algorithm is the most researched, widely used and mature algorithm among the point-based methods for LiDAR point cloud registration. In the ICP algorithm, the transformation between adjacent point clouds is iteratively calculated by minimizing a distance function. In [6], under the strong assumption that the number and relationship of the corresponding point pairs remain unchanged during the iteration process, the ICP algorithm was proven to be able to always monotonically converge to a local minimum. Researchers have gradually proposed many improved ICP algorithms to improve the accuracy, efficiency and robustness of the point cloud registration algorithm. Ref. [12] proposed a “point-to-point” ICP algorithm, which searches the corresponding relationship from the geometric closest point in the 3D shape. Ref. [13] introduces a “point-to-plane” ICP algorithm, which can be used for distance data by estimating the target model as a plane. Ref. [14] proposed the P2Pl-ICP algorithm, which uses the point-to-point distance as the error measure instead of the point-to-point distance to improve the robustness of the algorithm. Ref. [15] proposed the generalized ICP (GICP) algorithm. The core idea of this algorithm is to use the local continuity of the point cloud surface, approximate the surface shape around the point to a plane piece, and consider the noise model of the sensor, which can effectively reduce the impact of mismatching. Although this method shows strong effectiveness and robustness among many improved ICP algorithms, it is not as effective as the ICP algorithm in outdoor scenes [16]. To solve the problem of scenes with different reflection characteristics and the same geometric shape, reference [17] introduced the constraint of point cloud intensity information and proposed the Intensity-ICP method, which incorporated the intensity error measure into the objective function, adding a new constraint to the relative pose solution of point cloud registration and assigning a certain weight. Reference [18] proposed a further extension algorithm of the ICP algorithm, the CICP algorithm, which uses the estimated continuous posture to correct the distortion, and proposed a continuous-time trajectory estimation method for Ro-LiDAR SLAM. The scanning points are directly used to accurately estimate position and pose, and many points are required for stable registration. This method has the advantage of high matching accuracy, but it also has the problem of low computational efficiency.

### 2.2. Distribution-Based Method

The NDT algorithm is a typical distribution-based method that was first proposed in 2D LiDAR point cloud registration [7]. The LiDAR point cloud is represented by a group of Gaussian distributions with different probability density functions. To avoid the problem of incorrect correlation between data, the method gives the segmented smooth normal distribution representation of laser scanning data. The biggest advantage is that a clear correspondence between features or two points does not need to be established. Therefore, the algorithm has good robustness. Ref. [19] used real mine tests to prove that NDT has stronger adaptability, better accuracy and robustness than ICP. However, this method relies on NDT scanning registration for positioning and mapping. With the increase in the registration process, LiDAR odometry inevitably accumulates errors. Ref. [20] proposes a P2D-NDT scanning matching method, which extends the NDT method from 2D to 3D. The algorithm divides the reference frame LiDAR point cloud into small 3D grid cubes, calculates the probability density function of each grid using the shape of the internal contained points, and solves the relative pose transformation problem by maximizing the points in the current frame LiDAR scanning to the reference frame surface [21]. Ref. [22] proposes an extended version of the P2D-NDT algorithm, namely, the D2D-NDT scanning matching method, which represents two LiDAR point clouds with a normal distribution and introduces the selection of initial points and estimation of covariance in the iterative optimization algorithm. Therefore, compared with the P2D-NDT algorithm, the D2D-NDT algorithm improves the operational efficiency at the expense of robustness. The point cloud registration method of NDT has higher efficiency and a wider convergence range, but the lack of an initial value will also lead to the problem of local optimization.

### 2.3. Feature-Based Method

The feature-based method extracts some simple features from the LiDAR point cloud for feature matching to improve the efficiency of point cloud registration. Then, the extracted features are used to find the relative pose change relationship between points in point cloud registration. This simple feature can select points, lines, faces or combinations thereof. The point cloud registration method based on point features finds the corresponding points by extracting feature points, which is the most suitable for 2D point cloud registration. However, many feature descriptors are designed for application to specific environmental conditions. In [23], 2D scanning was used to match locally invariant CIF feature points extracted from LiDAR point cloud data. To solve the problem that point cloud registration is prone to fail in large-scale scenes, the corresponding algorithm is improved by extracting CIF feature points in [24]. Ref. [25] proposes a 2D point cloud registration method based on ICE, which uses multiple feature points, such as intersection points, corner points and wall endpoints, for point cloud registration. Ref. [26] proposed a point cloud registration method based on FLIRT and studied three feature detectors of LiDAR point clouds based on normal, curvature, distance, local shape context and two feature descriptions based on a β-grid. In conclusion, although a large number of point feature detection-based methods and feature descriptor-based algorithms [27,28] have been proposed for 3D LiDAR point cloud registration algorithms, most of them have difficulty performing efficient point cloud registration on 3D LiDAR point clouds because of the problems of algorithm accuracy, efficiency and robustness. The point cloud registration method based on line features has many simple and efficient line features in indoor scenes. The widely used segment-merge method is proposed in [29] for the point cloud registration method based on line features in 2D LiDAR point cloud data. The point cloud registration method based on face features compensates for the shortcomings of extracting point and line features and reasonably uses the extracted features for data association and point cloud matching by detecting a large number of plane or surface features in the region. For scenes containing curved objects, ref. [30] used voxel filters to uniformly downsample the original 3D LiDAR point cloud data and used points on the feature plane to register the point cloud, eliminating irrelevant interference outside the feature plane. Notably, Zhang et al. proposed a typical SLAM solution, called LOAM (LiDAR Odometry and Mapping), to obtain accurate results and reduce the computational complexity [8]. This method efficiently registered interframe point clouds by extracting the features of edges and planes in the environment and matching feature points to edge lines or planes. The system includes two independent threads: high-frequency odometry and low-frequency mapping. The former outputs LiDAR odometry via the point cloud registration of adjacent frame point clouds, and the latter outputs accurate attitude estimation by matching the current point cloud with the mapped frequency. However, feature-based methods have shown good performance in autonomous robot positioning and mapping, but this method results in errors due to the lack of geometric features or feature degradation scenarios, which will seriously affect the accuracy of point cloud registration.

The biggest difference between our method and other point cloud registration methods is that the traditional point cloud registration method for the special environment where the characteristics of coal mine roadway are degraded will often be trapped in the fast conversion of degraded corners or scenes, and it is difficult to show good point cloud registration performance. In order to adapt to the coal mine roadway environment, a better balance should be made on the registration accuracy, registration success rate and calculation complexity. We draw on the advantages of feature-based methods such as LOAM to extract simple feature information of point clouds from the environment to improve the efficiency and robustness of point cloud registration. At the same time, the IMU preintegration information is introduced to solve the problem of point cloud distortion correction, and the IMU error equation is constructed to improve the accuracy of point cloud registration. In addition, the RANSAC ground segmentation method is used to efficiently segment the point cloud and provide additional z-axis constraints for the ground mobile robot. The stability of point cloud registration is improved by eliminating unstable flawed points. A LiDAR point cloud registration method based on IMU preintegration is proposed, which provides a method reference for efficient point cloud registration of mobile robots in coal mine roadway and also lays a good foundation for the robustness of mobile robot LiDAR-SLAM.

## 3. Materials and Methods

### 3.1. System Framework

**Figure 1:**
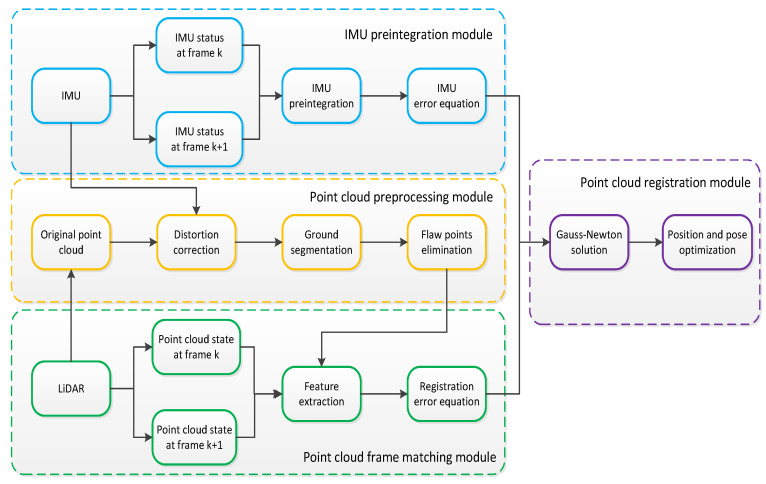
System framework diagram.

Our system input is 3D LiDAR and IMU sensor data. Our goal is to estimate a rigid transformation to align the optimal registration between two frame point clouds. The system framework of our proposed method consists of an IMU preintegration module, a point cloud preprocessing module, a point cloud frame matching module and a point cloud registration module. The system framework is shown in Figure 1.

- IMU preintegration module: the IMU preintegration model is constructed, and the IMU error equation is derived.
- Point cloud preprocessing module: the IMU linear interpolation method is used to correct the distortion of the point cloud to improve the quality of the original point cloud. Second, the ground segmentation method of RANSAC is used to segment the original point cloud into ground points and nonground points to provide additional ground constraints for z-axis displacement. Finally, the unstable flaw points are eliminated to improve the stability of point cloud registration.
- Point cloud frame matching module: feature corner points and plane points in the point cloud are extracted, and the point cloud registration error equation is constructed using the result of point cloud feature extraction between two frames.
- Point cloud registration module: the IMU error equation and the point cloud registration error equation are combined and solved by the Gauss–Newton method to minimize the error function and output the optimized position and pose.

### 3.2. IMU Preintegration Module

The IMU can obtain the three-axis acceleration and angular velocity information at the same time. By integrating them separately, the status information of the IMU coordinate system relative to the world coordinate system at any time can be obtained, including speed, position and pose. Since the PVQ integration results from the IMU coordinate system to the world coordinate system, the previous IMU measurements need to be reintegrated after each optimization update. To convert the integration model into the preintegration model, Formula (1) is introduced.

(1) $$q_{b_{t}}^{w}=q_{b_{i}}^{w}⊗q_{b_{t}}^{b_{i}}$$

In the above formula, $q_{b_{t}}^{b_{i}}$ represents the relative attitude change of IMU coordinate system from time *i* to time *t* (quaternion), $q_{b_{i}}^{w}$ represents the relative attitude change of IMU coordinate system from the world coordinate system to the moment *i* (quaternion), $q_{b_{t}}^{w}$ represents the relative attitude change of IMU coordinate system from the world coordinate system to the time *t* (quaternion).

The differential equation of the IMU measured value, the integral term in the PVQ integral formula from the ith moment to the jth moment, is converted from the attitude of the previous IMU coordinate system from the jth moment to the world coordinate system to the IMU preintegration component from the jth moment to the ith moment of the IMU coordinate system, which can be expressed as the following.

(2) $$p_{b_{j}}^{w}=p_{b_{i}}^{w}+v_{i}^{w}\Delta t-\frac{1}{2}g^{w}\Delta t^{2}+q_{b_{i}}^{w}\int \int_{t\in [i,j]}\left(q_{b_{t}}^{b_{i}}a^{b_{t}}\right)\delta t^{2}$$

(3) $$v_{j}^{w}=v_{i}^{w}-g^{w}\Delta t+q_{b_{i}}^{w}\int_{t\in [i,j]}\left(q_{b_{t}}^{b_{i}}a^{b_{t}}\right)\delta t$$

(4) $$q_{b_{j}}^{w}=q_{b_{i}}^{w}\int_{t\in [i,j]}q_{b_{t}}^{b_{i}}⊗\left[\begin{array}{c} 0\\ \frac{1}{2}\omega^{b_{t}} \end{array}\right]\delta t$$

In the above formula, the position, speed and attitude of the robot in the world coordinate system at time *i* are, respectively, expressed as $p_{b_{i}}^{w}$, $v_{b_{i}}^{w}$ and $q_{b_{i}}^{w}$, the acceleration and angular velocity of time *t* in the world coordinate system are, respectively, expressed as $a^{b_{t}}$ and $\omega^{b_{t}}$. The acceleration of gravity can be expressed as $g^{w}={\left[0\;0\;9.81\right]}^{T}$. The acceleration of gravity can be expressed as the position, velocity and attitude of the IMU coordinate system from the world coordinate system to the time *j*, respectively; $p_{b_{j}}^{w}$, $v_{b_{j}}^{w}$, $q_{b_{j}}^{w}$. ⊗ is expressed as quaternion multiplication.

The preproduct component is only related to the IMU measurement value and has no relationship with the state before time *i*. To concisely express the error equation of preintegration, the position preintegration component, $p_{b_{j}}^{b_{i}}$; velocity preintegration component, $v_{b_{j}}^{b_{i}}$; and pose preintegration component, $q_{b_{j}}^{b_{i}}$, are recorded from *i*th to *j*th (quaternion representation).

(5) $$p_{b_{j}}^{b_{i}}=\int \int_{t\in [i,j]}\left(q_{b_{t}}^{b_{i}}a^{b_{t}}\right)\delta t^{2}$$

(6) $$v_{b_{j}}^{b_{i}}=\int_{t\in [i,j]}\left(q_{b_{t}}^{b_{i}}a^{b_{t}}\right)\delta t$$

(7) $$q_{b_{j}}^{b_{i}}=\int_{t\in [i,j]}q_{b_{t}}^{b_{i}}⊗\left[\begin{array}{c} 0\\ \frac{1}{2}\omega^{b_{t}} \end{array}\right]\delta t$$

To constrain the preintegration state variables between two moments, including position error, velocity error, pose error, acceleration bias error and gyro bias error, the IMU preintegration component within a period of time is constructed as the measured value, and the error calculation formula is as follows.

(8) $$E_{\left(i,j\right)}^{B}={\left[\begin{array}{c} r_{p}\\ r_{v}\\ r_{q}\\ r_{ba}\\ r_{bg} \end{array}\right]}_{15\times 1}=\left[\begin{array}{c} q_{w}^{b_{i}}(p_{b_{j}}^{w}-p_{b_{i}}^{w}-v_{i}^{w}\Delta t+\frac{1}{2}g^{w}\Delta t^{2})-p_{b_{j}}^{b_{i}}\\ q_{w}^{b_{i}}(v_{j}^{w}-v_{i}^{w}+g^{w}\Delta t)-v_{b_{j}}^{b_{i}}\\ 2{\left[q_{b_{i}}^{b_{j}}⊗(q_{w}^{b_{i}}⊗q_{b_{j}}^{w})\right]}_{xyz}\\ b_{j}^{a}-b_{i}^{a}\\ b_{j}^{g}-b_{i}^{g} \end{array}\right]$$

In the above formula, $r_{p}$ represents the preintegration position error, $r_{v}$ represents the preintegration velocity error, $r_{q}$ represents the preintegration attitude error, $r_{ba}$ represents the IMU acceleration bias error and $r_{bg}$ represents the IMU screw torque bias error. Among the errors, displacement, velocity, and offset are directly subtracted. Only the attitude error is the rotation error of the quaternion ${\left[•\right]}_{xyz}$. represents the three-dimensional vector and consists of the imaginary part of quaternion (x,y,z).

### 3.3. Point Cloud Preprocessing Module

#### 3.3.1. LiDAR Point Cloud Distortion Correction Based on IMU Linear Interpolation

To solve the problem of LiDAR motion distortion, we use the IMU linear interpolation method to compensate for the motion of the current frame point cloud. In the process of LiDAR scanning, each LiDAR point has a unique timestamp. Assuming that the current frame point cloud acquisition process meets the uniform motion model, IMU integration can quickly obtain the pose at the current time. The IMU data from the beginning to the end of the current LiDAR frame are used to calculate the rotation increment, and IMU preintegration is used to calculate the translation increment. Then, the LiDAR point at each time of the frame is corrected for motion distortion using the pose increment relative to the beginning of the LiDAR frame. The motion state of each point is estimated in the current frame point cloud, the coordinate system from the current LiDAR point is transformed to the starting LiDAR point, the motion change of the laser radar is calculated during the acquisition process, and the amount of motion on the corresponding LiDAR point is compensated for to correct for the distortion of the point cloud.

**Figure 2:**
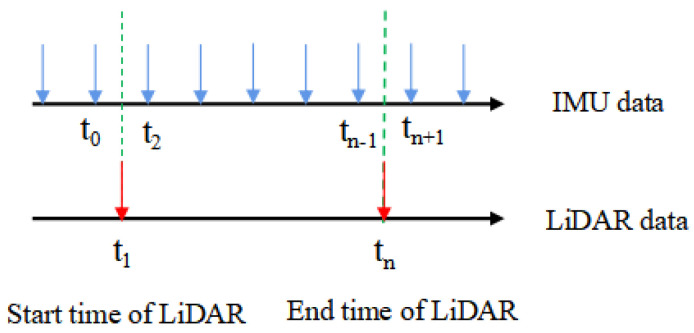
Schematic diagram of linear interpolation of IMU data.

Due to the different sampling frequencies of IMU and LiDAR point cloud data, the sampling time of the algorithm for two different types of sensors is not uniform. The acquired LiDAR point cloud and IMU measurement value must first be synchronized in time, and the relative motion of the LiDAR from the first point to the last point of the current frame point cloud can then be obtained by using the IMU preintegration, combined with the acquisition time of each point in the current frame point cloud data. Each point in the current frame point cloud is converted to the LiDAR coordinate system of the first point to complete the distortion correction of the single frame LiDAR point cloud. The LiDAR point cloud data at the current time and the last two IMU measurements are used for linear interpolation to achieve time registration between the LiDAR point cloud data and IMU data. The IMU data interpolation process is shown in Figure 2.

The current frame LiDAR point clouds, $t_{1}$ and $t_{n}$, are represented as the scanning start time and end time, respectively; the current frame LiDAR point cloud scanning start time, $t_{1}$, corresponds to the nearest IMU start and end times, $t_{0}$ and $t_{2}$, respectively, and the current frame LiDAR point cloud scanning end time, $t_{n}$, corresponds to the nearest IMU start and end times, $t_{n-1}$ and $t_{n+1}$, respectively. The position and pose of IMU at time t are p and q, respectively. Then, linear interpolation is carried out based on the measured value of IMU to obtain the position and pose of $t_{n}$ at the end of LiDAR point cloud scanning in the current frame, which can be expressed as follows.

(9) $$p_{n}=\frac{t_{n}-t_{n-1}}{t_{n+1}-t_{n-1}}•p_{n-1}+\frac{t_{n+1}-t_{n}}{t_{n+1}-t_{n-1}}•p_{n+1}$$

(10) $$q_{n}=\frac{t_{n}-t_{n-1}}{t_{n+1}-t_{n-1}}•q_{n-1}+\frac{t_{n+1}-t_{n}}{t_{n+1}-t_{n-1}}•q_{n+1}$$

The position change of IMU can be expressed as δp, the attitude change of IMU can be expressed as δq, and the position change of IMU can be expressed as the following.

(11) $$\delta p=p_{n}-p_{1}$$

(12) $$\delta q=q_{1}^{-1}•q_{n}$$

The attitude quaternion is transformed from the Rodriguez formula into a transformation matrix, $\delta T_{B}$, which consists of a rotation matrix and translation vector in homogeneous coordinates. Assuming that the external parameter matrix, $T_{B}^{L}$, from the IMU coordinate system to the LiDAR coordinate system has been obtained through external parameter calibration, there is a point $X_{i}$ in the original point cloud of the current frame before distortion correction, and the point is converted to the coordinate system where the LiDAR scanning starting point of the current frame is located through distortion correction, which is X, as shown in the following table.

(13) $$X=\frac{cur-start}{end-start}•T_{B}^{L}•\delta T_{B}•X_{i}$$

In the above formula, the horizontal angle of the current point $X_{i}$ is expressed as cur, the horizontal angle of the current frame scan start point is expressed as start, and the horizontal angle of the current frame scan end point is expressed as end. The points in the point cloud are traversed in turn, and all LiDAR points are converted to the coordinate system where the LiDAR scanning start point of the current frame is located, thus completing the distortion correction of the single frame point cloud in motion.

#### 3.3.2. Ground Segmentation Based on RANSAC

In the open source algorithm of hdl-glaph-slam, the ground constraint constructed also uses the RANSAC ground segmentation method. When the ground in the environment is a plane, it can be used as very effective information to constrain the elevation error, and those who have done laser slam know that in the absence of GPS, the elevation error is the main error item. Of course, it cannot be used when the ground is non-planar, but this cannot be taken as its disadvantage. Instead, it should be decided whether to enable this function according to the environment. In our method, because there is no GPS signal under the coal mine roadway, we introduced the RANSAC algorithm to segment the ground for two purposes: First, for the ground mobile robot, the ground point cloud often accounts for one-third of the total point cloud. The ground plane point cloud is separated, which greatly reduces the calculation time of feature extraction in the later stage. Second, when the ground robot works in different space areas, adding ground constraints can provide additional constraints for the z-axis displacement of key frame nodes and reduce the cumulative error, as inspired by the literature [31]. Completing ground segmentation in the shortest time is a key problem, and a robust estimation method with fewer iterations and strong antinoise ability should be selected. This paper selects a random sample consensus (RANSAC) [32] to solve the above problems.

According to the basic principle of RANSAC, three points are randomly selected from each frame of the point cloud to obtain a plane. The commonly used plane equation is *ax* + *by* + *cz* = *d*, where *a*^2^ + *b*^2^ + *c*^2^ = 1, *d* > 0, (*a*, *b*, *c*) is the plane normal vector, and the distance between the LiDAR sensor to the plane is denoted as *d*. These four parameters can determine a plane. The specific steps are as follows:

1. Select three points $P_{1}(x_{1},y_{1},z_{1})$, $P_{2}(x_{2},y_{2},z_{2})$, $P_{3}(x_{3},y_{3},z_{3})$ randomly from point cloud data *P*.
2. Plane *S* is determined accoding to three points $P_{1}(x_{1},y_{1},z_{1})$, $P_{2}(x_{2},y_{2},z_{2})$ and $P_{3}(x_{3},y_{3},z_{3})$. The values of parameters *a*, *b*, *c* and *d* are determined by Formula (14).
  (14) $$\left\{\begin{array}{l} ax_{1}+by_{1}+cz_{1}=d\\ ax_{2}+by_{2}+cz_{2}=d\\ ax_{3}+by_{3}+cz_{3}=d \end{array}\right.$$
3. Calculate the number of points on plane *S* in point cloud data *P*. Set the plane thickness, ε (point to plane distance threshold), and calculate the distance between any point $P_{i}(x_{i},y_{i},z_{i})$ in *P* and plane *S*, where $d_{i}$ is calculated by Formula (15).

(15) $$d_{i}=\left|ax_{i}+by_{i}+cz_{i}-d\right|$$

Then, calculate the number of points that meet the requirements of $d_{i}<\varepsilon$, and record its score as plane *S*.

4. Repeat the above steps to sample *K* times and select Plane $S_{x}$ with the highest score.

(16) $$1-1-C_{m}^{n}C_{m-1}^{n-1}C_{m-2}^{n-2}{}^{K}=\varphi$$

In Formula (16), the number of points in the laser point cloud *P* is expressed as *m*, the number of points on the plane *S* is expressed as *n*, and the probability of selecting the base plane after *K* times of sampling is expressed as φ. Since m and n are large, approximate calculation is used here, and the simplified formula is as follows:

(17) $$1-1-1-\tau^{3K}=\varphi$$

In Equation (17), τ is the probability that the point is outside of plane $S_{x}$, and *K* is obtained after simplification, as shown in Equation (18).

(18) $$K=\frac{\log(1-\varphi )}{\log1-1-\tau^{3}}$$

5. The selected ground plane data were refitted to obtain ground plane parameters with less error.

#### 3.3.3. Flaw Point Elimination

**Figure 3:**
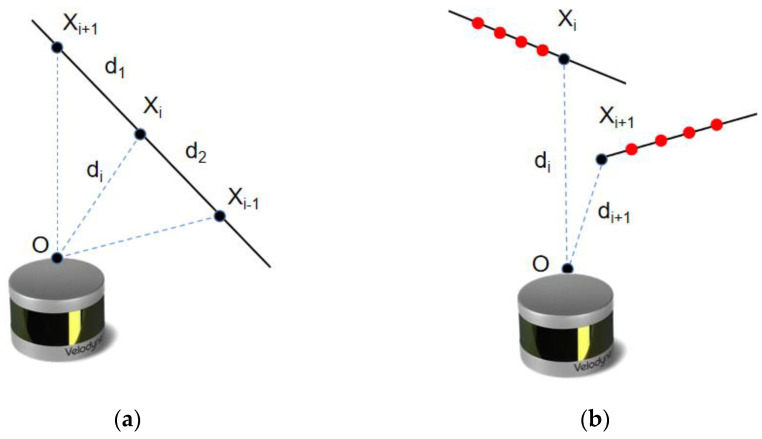
Unstable flaw points. (**a**) Schematic diagram of parallel flaw points; (**b**) Schematic diagram of occluded flaw points.

Since not every point whose curvature value meets the screening criteria can be used as a feature point, its curvature value may meet the standard due to environmental factors, but the actual curvature value of the point will change due to the change in the angle of view, resulting in its loss of characteristics. Such points are called defects and should be eliminated. Unstable flaw points include parallel and occlusion points, as shown in the Figure 3.

If the wall plane is approximately parallel to the LiDAR scanning line in the process of LiDAR scanning, the plane feature point at the last moment will completely disappear when the mobile robot equipped with LiDAR continues to move until it is completely parallel to the laser ray because its continuous point set is in the same plane and combined with the curvature calculation formula. To improve the stability of point cloud registration, unstable parallel flaw points must be removed. The specific screening method is to calculate the distance between point $X_{i}$ and its adjacent points before and after, $d_{1}$ and $d_{2}$, and the linear distance $d_{i}$ from point $X_{i}$ to the LiDAR origin. When the parallelism of the LiDAR harness and the plane is higher, the ratio of $d_{1}$ and $d_{2}$ to $d_{i}$ will increase. When the ratio exceeds the set threshold, the point is considered defective. According to the parallelism characteristics of the LiDAR scanning plane, the distance between the selected point and its adjacent 3D points is calculated. A greater distance between the adjacent points corresponds to a smaller tangent angle between the plane of the candidate point and the LiDAR beam, and the point is then eliminated based on the set threshold.

During LiDAR scanning, two object boundaries may block each other. In this case, due to the occlusion of the angle of view, the points with small curvature will form a continuous point set with the occluding boundary, resulting in an inconsistency between the calculated curvature and the geometric characteristics of the robot. After the robot changes its posture and angle of view, its curvature value will inevitably significantly change, which does not meet the requirements for the stability of the geometric characteristics of the feature points. Therefore, such candidate points need to be eliminated.

The specific screening method is to take $X_{i}$ as the occluded point. When it is classified as a defect point, its five consecutive adjacent points will also lose the opportunity to be selected as a feature point because they are too close to the defect point. First, the distance $d_{i}$ and $d_{i+1}$ between the current point $X_{i}$ and its adjacent point $X_{i+1}$ are calculated from the LiDAR coordinate system; the distance $d_{\left(i,i+1\right)}$ between the two points is also calculated to then construct an isosceles triangle according to the size of $d_{i}$ and $d_{i+1}$. The angle between the vectors $X_{i}$ and $X_{i+1}$ is then calculated. When the value of the angle is lower than the set threshold, this point and its adjacent point can be considered to be on different planes and cannot be corner points. Therefore, the points can be eliminated.

### 3.4. Point Cloud Frame Matching Module

The feature extraction method we adopted is similar to the method introduced in LOAM [8]. First, edge points and plane points are selected by calculating the local smoothness, the residual of the LiDAR observation model is constructed, the corresponding edge line points and plane points in other key frames are selected, and feature corners and plane points are extracted. In addition, reflectivity is also used as an additional determinant. If the reflectivity of a point is different from the adjacent threshold, the point is also considered another edge point. The plane smoothness is calculated according to the curvature of the point as an index to extract the feature information of the current frame. Then, the curvature, *c*, of the evaluation local surface is determined, which can be expressed as the following.

(19) $$c=\frac{1}{|S|\cdot \left\|X_{\left(k,i\right)}^{L}\right\|}\left\|\sum_{j\in S,j\neq i}\left(X_{\left(k,i\right)}^{L}-X_{\left(k,j\right)}^{L}\right)\right\|$$

In the above formula, the number of points in the neighbourhood of the calculated point is expressed as S, the three-dimensional coordinates of point ith at time *k* under the LiDAR coordinate system are expressed as $X_{\left(k,i\right)}^{L}$, and the three-dimensional coordinates of point jth at time k under the LiDAR coordinate system are expressed as $X_{\left(k,j\right)}^{L}$.

The points in the scan are sorted according to the c value, and the point with the maximum c value is selected as the corner point; that is, the point on the sharp edge in 3D space has a large difference in size from the surrounding points, high curvature and high smoothness. The point with the minimum c value is selected as the plane point, that is, the point on the smooth plane in 3D space has a small difference in size with the surrounding points, low curvature and low smoothness.

In the local features extracted from the LiDAR point cloud, the points with relatively large curvature are used as feature corners. Considering that they are distributed on the edge of the object, the corresponding relationship of their construction is to find two points in the point cloud of the previous frame to form a line closest to the current feature point as a constraint relationship. The distance, $d_{e}$, from the corner point in the current frame, $t_{k}$, to the two points in the previous frame, $t_{k-1}$, to determine the straight line is used as the registration standard, and $d_{e}$ is calculated as follows.

(20) $$d_{e}=\frac{\left|\left(X_{\left(k,i\right)}^{L}-X_{\left(k-1,j\right)}^{L})\times (X_{\left(k,j\right)}^{L}-X_{\left(k-1,l\right)}^{L}\right)\right|}{\left|X_{\left(k-1,j\right)}^{L}-X_{\left(k-1,l\right)}^{L}\right|}$$

In the above formula, the coordinate of point *i* under the LiDAR coordinate system at time *k* is expressed as $X_{\left(k,i\right)}^{L}$, the coordinate of point *j* under the LiDAR coordinate system at time *k*−1 is expressed as $X_{\left(k-1,j\right)}^{L}$, and the coordinate of point *l* under the LiDAR coordinate system at time *k*−1 is expressed as $X_{\left(k-1,l\right)}^{L}$. The corresponding relationship of feature corners, where *j* and *l* are the two points closest to point *i* in the LiDAR coordinate system at time *k* under the LiDAR coordinate system at time *k*−1, and *j* and *l* are distributed on different scanning lines.

Similarly, in the local features of the point cloud, the extracted feature plane points are mainly concentrated on the plane with relatively low curvature. Therefore, considering that they are distributed in the plane part of the associated object, the corresponding relationship of their construction is to find the current feature point in the LiDAR point cloud of the last frame and the plane formed by the three closest points as a constraint relationship. The distance, $d_{p}$, from the plane point in the current frame, $t_{k}$, to the plane determined by the three points in the previous frame, $t_{k-1}$, is selected as the registration standard, and $d_{p}$ is calculated as follows.

(21) $$d_{p}=\frac{\left|\begin{array}{c} \left(X_{\left(k,i\right)}^{L}-X_{\left(k-1,j\right)}^{L}\right)\\ \left(X_{\left(k-1,j\right)}^{L}-X_{\left(k-1,l\right)}^{L})\times (X_{\left(k-1,j\right)}^{L}-X_{\left(k-1,m\right)}^{L}\right) \end{array}\right|}{\left|\left(X_{\left(k-1,j\right)}^{L}-X_{\left(k-1,l\right)}^{L})\times (X_{\left(k-1,j\right)}^{L}-X_{\left(k-1,m\right)}^{L}\right)\right|}$$

In the above formula, the coordinate of point *i* under the LiDAR coordinate system at time *k* is expressed as $X_{\left(k,i\right)}^{L}$, the coordinate of point *j* under the LiDAR coordinate system at time *k*−1 is expressed as $X_{\left(k-1,j\right)}^{L}$, the coordinate of point *l* under the LiDAR coordinate system at time *k*−1 is expressed as $X_{\left(k-1,l\right)}^{L}$, and the coordinate of point m under the LiDAR coordinate system at time *k*−1 is expressed as $X_{\left(k-1,m\right)}^{L}$. Where *j*, *l*, and *m* are the three closest points under the LiDAR coordinate system at *k*−1 to point *i* under the laser radar coordinate system at *k*, *j* and *l* are distributed on the same scan line, and *m* is distributed on the same scan line that is not the same as *j* and *l*.

The extracted feature corner points and feature plane points are represented as $F_{ei}$ and $F_{pi}$ in the LiDAR scanning features at the ith frame, respectively. These features form a set of all extracted features in the ith frame, which can be expressed as $F_{i}=\left\{F_{ei},F_{pi}\right\}$. The above two distances are taken as the optimization objects of the objective function to construct the optimization equation.

(22) $$f_{e}(X_{\left(k+1,F_{ei}\right)}^{L},T_{k+1}^{L})=d_{\left(e,i\right)}$$

(23) $$f_{p}(X_{\left(k+1{,}_{F_{pi}}\right)}^{L},T_{k+1}^{L})=d_{\left(p,i\right)}$$

In the above formula, point $X_{\left(k+1,F_{ei}\right)}^{L}$ is the point in the feature corner point set, point $X_{\left(k+1,F_{pi}\right)}^{L}$ is the point in the feature plane point set, and point $T_{k+1}^{L}$ is the pose transformation of the point cloud at time *k* + 1. Through nonlinear optimization, $T_{k+1}^{L}$, which minimizes the residual error of the objective function, f(•), is obtained as the optimal scan-to-scan pose estimation of the current frame.

The distance, d(e,i), from the feature point to the line and the distance, d(p,i), from the feature point to the plane are used as the observation values of the registration error of the two frames. The error function calculation formula of point cloud registration can be expressed as the following.

(24) $$E_{\left(k,F_{i}\right)}^{L}=\sum_{i=1}^{F_{ei}}d(e,i)+\sum_{i=1}^{{}_{F_{pi}}}d(p,i)$$

In the above formula, the feature corner points extracted from the point cloud are expressed as $F_{ei}$, and the feature plane points extracted from the point cloud are expressed as $F_{pi}$.

### 3.5. Point Cloud Registration Module

Because the point cloud of the LiDAR is too sparse, it is impossible to observe the same point in the front and back frame point clouds, so the point-to-point ICP used for the LiDAR point cloud matching has poor effect. However, in fact, the points in the original point cloud can also be considered to be distributed on a plane. According to this assumption, the surface-to-surface ICP algorithm, also known as the generalized ICP algorithm (GICP), is proposed. The core idea of GICP is a probability model for the ICP minimization step. The standard Euclidean distance is used to replace the probability measure to calculate the corresponding relationship, thus maintaining the main advantages of GICP compared with other full probability technologies. However, in view of the actual situation of point cloud feature degradation in the coal mine roadway environment, even the point cloud registration method using GICP also shows great disadvantages.

Given two frame point clouds $X=\left\{x_{i}\in {\mathbb{R}}^{3}\left|\;i=1,\ldots ,M\right.\right\}$ and $Y=\left\{y_{i}\in {\mathbb{R}}^{3}\left|\;i=1,\ldots ,N\right.\right\}$, our goal is to estimate a rigid transformation $T=\left\{R,t\right\}$ to align the current frame point cloud with the target frame point cloud [33], where R∈SO(3) is a rotation matrix and $t\in {\mathbb{R}}^{3}$ is a translation vector. These two clouds can have different numbers of points, namely, M≠N. The transformation relationship of the rigidity change can be expressed as the following.

(25) $$\underset{R,t}{\min}{\sum \left\|R\cdot x_{i}+t-y_{i}\right\|}_{2}^{2}$$

Because the error constraint of the LiDAR point cloud registration of the ground mobile robot cannot provide high-precision state estimation of the roll angle and pitch angle, resulting in significant deviation of the fused point cloud scene in the Z-axis direction, IMU sensor data are introduced to further constrain the LiDAR motion state estimation and build a jointly optimized error function. Assuming that the deterministic error and random error of IMU have been eliminated by calibration, IMU error only comes from the position error, velocity error, pose error, accelerometer bias error and gyroscope bias error of preintegration. For the convenience of calculation, assuming that the origin of the world coordinate system coincides with the origin of the IMU starting time coordinate system, the IMU state variable $X_{k}^{B}$ at time k is Formula (26), and the IMU state variable $X_{k}^{L}$ under the LiDAR coordinate system is Formula (27).

(26) $$X_{k}^{B}=\left[p_{k}^{B},v_{k}^{B},q_{k}^{B},b_{k}\right]$$

(27) $$X_{k}^{L}=T_{B}^{L}X_{k}^{B}=\left[p_{k}^{L},v_{k}^{L},q_{k}^{L},b_{k}\right]$$

The state variable $x(t_{x},t_{y},t_{z},\theta_{x},\theta_{y},\theta_{z})$ is defined; then, the position and attitude under the LiDAR coordinate system at moment $t_{k}$ are $T_{k}^{L}$, which can be expressed as follows.

(28) $$T_{k}^{L}=\left[t_{x},t_{y},t_{z},\theta_{x},\theta_{y},\theta_{z}\right]=(p_{k}^{L},q_{k}^{L})$$

Since the LiDAR pose definition only contains position and pose information, the IMU preintegration error is simplified to include only IMU position error and pose error. The state error of the IMU coordinate system is transformed into the LiDAR coordinate system by Formula (27), and the overall error function is constructed by combining the point cloud registration error to optimize the LiDAR pose. Construct the joint optimization error function $F(T_{k}^{L})$ of LiDAR and IMU fusion, which can be expressed as follows.

(29) $$F(T_{k}^{L})=\frac{1}{2}{\left\|E_{\left(k,F_{i}\right)}^{L}+E_{\left(k,k+1\right)}^{L}\right\|}^{2}$$

In the above formula, the LiDAR pose at time k can be expressed as $T_{k}^{L}$, the IMU pose error under the LiDAR coordinate system from time *k* to time *k* + 1 can be expressed as $E_{\left(k,k+1\right)}^{L}$ (see Formula (8) and (27)), and the LiDAR point cloud scan to scan registration error from time *k* to time *k* + 1 can be expressed as $E_{\left(k,F_{i}\right)}^{L}$ (see Formula (24)).

The Gauss–Newton nonlinear optimization method is used to minimize the error function. Its core idea is to carry out the first-order Taylor expansion of the cost function f(x+Δx) and then construct the derivative of the quadratic error function. The reverse direction of the derivative is the gradient descending direction to iteratively solve the optimal state variable. The main formula is defined as follows.

(30) $$\Delta x^{*}=\arg\min\frac{1}{2}{\left\|f(x)+J{\left(x\right)}^{T}\Delta x\right\|}^{2}$$

In the above equation, J(x) is the Jacobi matrix, and the derivative of Δx such that the derivative of the error function is 0 yields the following.

(31) $$J(x)J{\left(x\right)}^{T}\Delta x=-J(x)f(x)$$

When Δx is sufficiently small, the iteration is stopped; otherwise, $x_{k+1}=x_{k}+\Delta x$ uses the update of the current status to continue the iteration. The core of such optimization problems is to solve the Jacobian matrix, J(x). The solution of the Jacobian matrix can be expressed as the following.

(32) $$J(x)=\left[\begin{array}{l} \frac{\partial x_{k+1}}{\partial t_{x}}\frac{\partial x_{k+1}}{\partial t_{y}}\frac{\partial x_{k+1}}{\partial t_{z}}\frac{\partial x_{k+1}}{\partial \theta_{x}}\frac{\partial x_{k+1}}{\partial \theta_{y}}\frac{\partial x_{k+1}}{\partial \theta_{z}}\\ \frac{\partial y_{k+1}}{\partial t_{x}}\frac{\partial y_{k+1}}{\partial t_{y}}\frac{\partial y_{k+1}}{\partial t_{z}}\frac{\partial y_{k+1}}{\partial \theta_{x}}\frac{\partial y_{k+1}}{\partial \theta_{y}}\frac{\partial y_{k+1}}{\partial \theta_{z}}\\ \frac{\partial z_{k+1}}{\partial t_{x}}\frac{\partial z_{k+1}}{\partial t_{y}}\frac{\partial z_{k+1}}{\partial t_{z}}\frac{\partial z_{k+1}}{\partial \theta_{x}}\frac{\partial z_{k+1}}{\partial \theta_{y}}\frac{\partial z_{k+1}}{\partial \theta_{z}} \end{array}\right]$$

The above Gauss–Newton algorithm can be used to optimize the solution of the state variables of the pose. The point cloud data at time *k* + 1 can be registered to the coordinate system of the point cloud at time *k* through coordinate transformation, and the relative motion of adjacent frames can be completed for state estimation. The pseudo-code of the LiDAR point cloud registration algorithm based on IMU preintegration is shown in Algorithm 1.


**Algorithm 1: LiDAR point cloud registration algorithm**
Input: IMU state variables at time k, point cloud {X} at frame k and point cloud {Y} at frame *k* + 1Output: State variable $x(t_{x},t_{y},t_{z},\theta_{x},\theta_{y},\theta_{z})$1: Solve PVQ between two IMU moments;2: Construct the error function $E_{\left(i,j\right)}^{B}$ of preintegration state quantity;3: The IMU state variable at time k is transferred to $X_{k}^{L}=T_{B}^{L}X_{k}^{B}$ in the LiDAR coordinate system;4: Extract point cloud feature corner point $F_{ei}$ and feature plane point $F_{pi}$;5: Construct the point cloud registration error function $E_{\left(k,F_{i}\right)}^{L}$ between two frames;6: Joint optimization error function $F(T_{k}^{L})=\frac{1}{2}{\left\|E_{\left(k,F_{i}\right)}^{L}+E_{\left(k,k+1\right)}^{L}\right\|}^{2}$;7: First order Taylor expansion according to f(x+Δx);8: Solve the optimal state variable $\Delta x^{*}=\arg\min\frac{1}{2}{\left\|f(x)+J{\left(x\right)}^{T}\Delta x\right\|}^{2}$;9: Find Δx and make $\Delta x^{*}$ reach the minimum;10: If Δx is small enough do the following:11:  Solve Jacobian matrix J(x);12:  Return state variable $x(t_{x},t_{y},t_{z},\theta_{x},\theta_{y},\theta_{z})$;13: Otherwise14: Update current status with $x_{k+1}=x_{k}+\Delta x$;15: Return to step 9;16: end.

## 4. Results and Discussion

**Figure 4:**
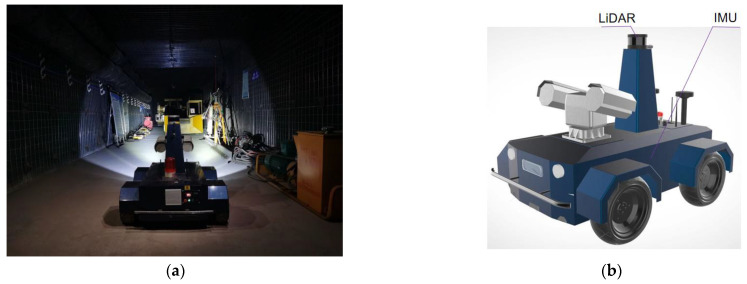
Wheeled mobile robot. (**a**) Physical picture of mobile robot; (**b**) 3D model of mobile robot.

We carried out a series of experiments to verify the proposed LiDAR point cloud registration method and compared it with other traditional point cloud registration methods. The hardware platform we used is a wheeled mobile robot with sensors and a computer, as shown in Figure 4. The sensor used in the experiment here includes a Velodyne VLP-16 LiDAR and a hipnuc-CH110 IMU. The LiDAR has a sampling frequency of 10 Hz, while the IMU has a sampling frequency of 200 Hz. Main parameters of the robot, overall dimensions: 1150 × 800 × 900 (L × W × H), running speed: 3 km/h, weight: 130 kg, maximum climbing angle: 30°. The airborne computer is an Intel Core i7 with a main frequency of 2.7 GHz, eight cores and 16 G memory, and all algorithms were implemented in C++ and executed on an Ubuntu 18.04 system using the medoic version of ROS.

First, the original point cloud was verified to be corrected by point cloud distortion to remove the point cloud distortion caused by the translation and rotation of the robot carrier. Second, the performance of four different algorithms for point cloud registration on public datasets and self-collected datasets was verified (including registration accuracy, registration success rate and calculation time). Finally, four groups of self-mining datasets were verified in the coal mine roadway environment, and the LiDAR odometry based on GICP and the LiDAR odometry fused with IMU preintegration were constructed for comparative analysis. The length error and absolute position and pose error of four groups of different trajectories were evaluated.

### 4.1. Point Cloud Distortion Correction Experiment

The point cloud distortion correction experiment shows two typical point cloud distortion correction cases, which are caused by translation and rotation.

#### 4.1.1. Point Cloud Distortion Caused by Translation

**Figure 5:**
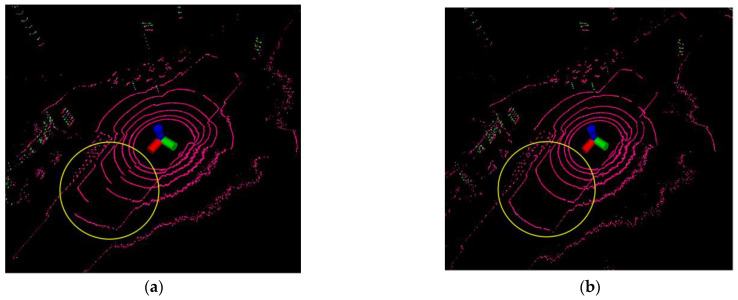
Point cloud distortion caused by translation. (**a**) Before distortion correction; (**b**) After distortion correction.

When the robot is equipped with LiDAR driving normally, due to the working principle of mechanical rotating LiDAR, it does not finish collecting the point cloud data for one week of the current frame at the same moment, which will cause point cloud distortion, as shown in the upper left corner of Figure 5. The point cloud inside the yellow circle should be continuous, but it is fragmented due to motion distortion. Therefore, we use the IMU linear interpolation point cloud distortion correction algorithm to correct the distortion of the original point cloud and obtain the distortion-corrected point cloud. As shown in the upper right corner of the figure, the point cloud inside the yellow circle is changed from a fragmented state to a continuous state after the point cloud distortion correction, and the result shows that the distortion correction algorithm is effective for removing the distortion caused by translation.

#### 4.1.2. Point Cloud Distortion Caused by Rotation

**Figure 6:**
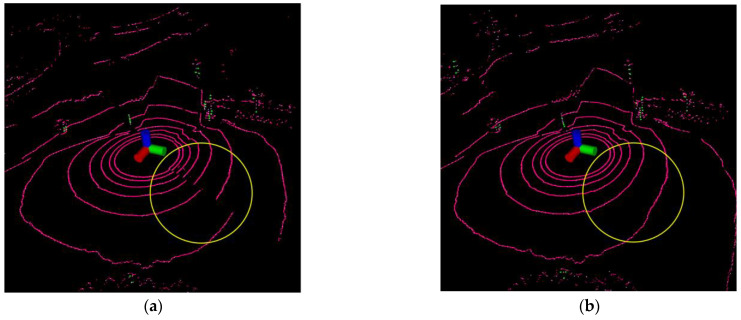
Point cloud distortion caused by rotation. (**a**) Before distortion correction; (**b**) After distortion correction.

When the robot rotation direction is opposite to the LiDAR motor rotation direction, the point cloud scanned by LiDAR for one week is missing the environmental information of a certain angle due to the effect of motion distortion, which will cause point cloud distortion, as shown in the lower-left corner of the Figure 6. The point cloud inside the yellow circle should be continuous, but it is markedly fragmented due to motion distortion. Therefore, we used the IMU linear interpolation point cloud distortion-correction algorithm to correct the distortion of the original point cloud and obtain the distortion-corrected point cloud. As shown in the bottom right corner of the figure, the point cloud inside the yellow circle is changed from a fragmented state to a continuous state after the point cloud distortion correction, and the results show that the distortion correction algorithm is effective in removing the distortion caused by rotation.

### 4.2. Point Cloud Preprocessing Experiment

**Figure 7:**
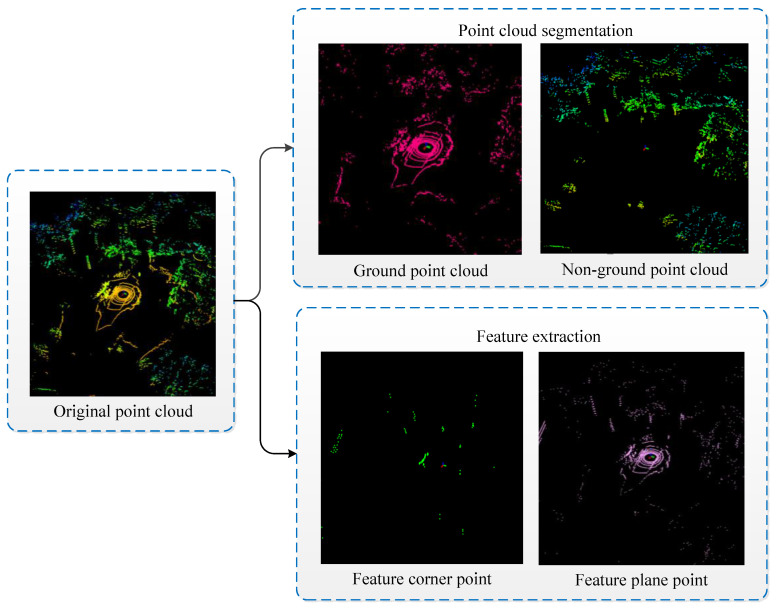
Point cloud preprocessing experiment.

The point cloud preprocessing experiment is mainly divided into two parts: ground segmentation and feature extraction, as shown in Figure 7.

The former uses RANSAC’s ground extraction algorithm for ground segmentation, which divides the original point cloud into ground points and nonground points, with the red ring line representing the ground points extracted from the current frame and the green point cloud representing the nonground points extracted from the current frame. Meanwhile, the original point cloud is processed by the ground segmentation algorithm, which can greatly improve the computational efficiency of feature extraction. Since the original point cloud is dense and has more unstable flaw points, part of the point cloud is cluttered, and the structural features of the environment are not obvious. Therefore, the flaw points will first be eliminated, and the feature point cloud will then be extracted from the point cloud to obtain the feature point cloud, where the green points represent the feature corner points for feature extraction and the pink points represent the feature plane points for feature extraction. Meanwhile, the feature point cloud is relatively sparse, and only some feature corner points and feature plane points with stronger structural features are retained, while the unstable feature points have all been eliminated.

### 4.3. Point Cloud Registration Experiment

**Figure 8:**
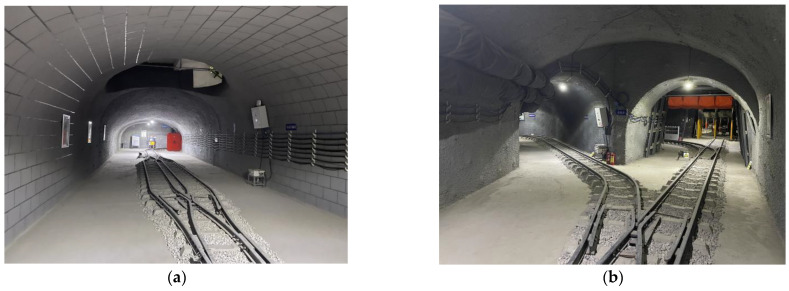
Partial environment of coal mine roadway. (**a**) Coal mine roadway; (**b**) Coal mine multi-roadway intersection.

The original point cloud undergoes the process of point cloud distortion correction, ground segmentation, flaw point rejection and feature extraction, which lays a good foundation for the next point cloud registration. To verify the performance of our proposed method (including registration accuracy, registration success rate and computational efficiency) and ensure the rationality of the experiment, we adopted the cross-validation method of public datasets and self-harvested datasets for comparative validation. To verify whether different line numbers of LiDAR will have an impact on the experimental results, we used the 64-line velodyne public dataset KITTI and the 16-line velodyne public dataset Park to conduct comparison experiments between different point cloud registration algorithms, and the true value is provided by the high-precision GPS of the dataset. Meanwhile, to further test the special environment of the coal mine tunnel studied in this paper, we used our own wheeled mobile robot system equipped with VLP-16 to collect point cloud data in the coal mine tunnel environment for point cloud registration experiments (note: in this experiment, only LiDAR and IMU are used for data collection) and the true value is provided by the state estimation of 6DOF by the high-precision laser SLAM algorithm [34] as a reference, as shown in Figure 8. To prove its superiority, our proposed LiDAR with IMU method is compared with the traditional methods, i.e., GICP [16], ICP [16] and NDT [13] algorithms, for quantitative analysis, a registration success rate test and a computational complexity test, respectively.

#### 4.3.1. Quantitative Analysis

**Figure 9:**
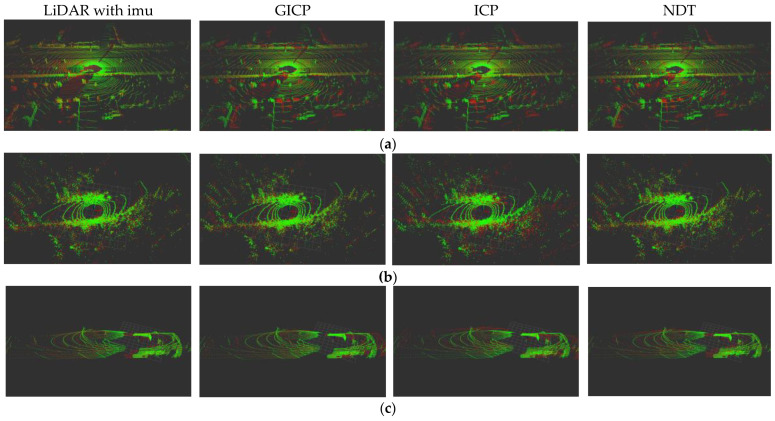
Display of point cloud registration results of different registration algorithms. (**a**) KITTI datasets; (**b**) Park datasets; (**c**) Coal mine roadway datasets.

To verify the difference between the performance of our point cloud registration algorithm (LiDAR with imu) and the traditional algorithm, we performed cross-validation on both public and self-harvested datasets. KITTI datasets is the largest automatic driving scene evaluation datasets at present, including data collected by many different types of sensors. Among them, we pay special attention to Velodyne 64-line 3D LiDAR, RTK GPS navigation system and 6-axis 100 Hz IMU measurement data. The Park datasets come from the VLP-16 datasets collected by the UGV robot Clearpath Jackal in LIO-SAM work. The robot is equipped with a VLP-16 3D laser radar, its rotation rate is set at 10 Hz, and a 9-axis IMU is built in. The success of point cloud registration depends on the overlapping area of the aligned point clouds. The overlap points were judged by the distance between matched counterparts as the closest points between point clouds. In this study, the distance threshold was set to 10 cm, and an overlap area of more than 80% represented successful registration. To compare the performance of different point cloud registration algorithms, we randomly selected 2389 and 1503 pairs of laser point cloud data from the KITTI and Park datasets and selected 528 pairs of LiDAR point cloud data from the coal mine roadway self-mining datasets for the experiment. We selected two pairs of point clouds with good performance in three different datasets to display the effect of point cloud registration experiments, as shown in Figure 9. The green point cloud represents the target frame point cloud, and the red point cloud represents the current frame point cloud.

To further improve the comparative experiment, we calculated statistics on the average value of the absolute position and pose changes (3DOF translation and 3DOF rotation) of the point cloud registration success of different algorithms under the three datasets, as shown in Table 1. The best results of the data obtained below are shown in bold.

The statistical results as shown in Table 1 that the proposed algorithm has smaller translation and rotation errors than the other three algorithms in the point cloud registration experiment under the same conditions of different datasets. Therefore, our proposed algorithm improved the point cloud registration accuracy.

#### 4.3.2. Comparison of Point Cloud Registration Success Rate

**Figure 10:**
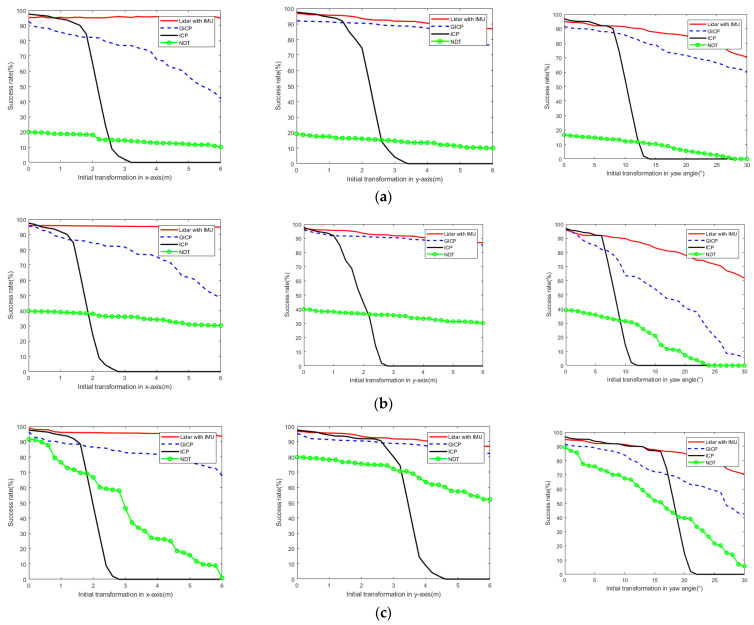
Success rate of different point cloud registration algorithms; (**left**) initial transformation in x-axis direction, (**middle**) y-axis direction, (**right**) initial yaw angle. (**a**) KITTI datasets; (**b**) Park datasets; (**c**) Coal mine roadway datasets.

As the experiment progressed, we found that the success rate of point cloud registration depended on the initial position of the point cloud. Therefore, to explore the difference in the success rate of different point cloud registration algorithms, we counted the impact on the success rate of point cloud registration when the x-axis, y-axis and yaw angle changed in different scenarios. The initial absolute pose range was set to the following: x-axis (−6–+6 m), y-axis (−6–+6 m), and θ (−30–+30°). LiDAR point cloud data were paired to test the point cloud registration success rate, as shown in Figure 10, where the directions of the x-axis and the y-axis represent the forward direction and the transverse direction of the robot, respectively, and θ represents the heading angle of the robot.

When considering the motion of the ground mobile robot, large changes were observed in the position and pose in the x-axis and yaw angles. Therefore, we should focus on the change in the point cloud registration success rate with increasing x-axis and yaw angles. As shown in Figure 9, the LiDAR with IMU method proposed by us in different scenarios (especially under the influence of large initial transformation conditions) had a higher success rate of point cloud registration than other traditional algorithms. Although the GICP algorithm is similar to the curve of our proposed method, the success rate significantly decreased with increasing x-axis directions and yaw angles. However, the stability of the ICP algorithm was poor. When the initial transformation exceeded a certain critical value along the x-axis, y-axis and yaw angle, the success rate of point cloud matching sharply decreased. Notably, the overall success rate of the NDT algorithm is not high and only shows acceptable results for small initial transformations. However, as the initial transformations or the degradation of point cloud features increased, the success rate of point cloud registration significantly decreased, especially in the environment of coal mine roadway feature degradation. In conclusion, the point cloud registration method we proposed has a higher success rate than other traditional methods, especially when the initial change with the x-axis and yaw angle is large.

#### 4.3.3. Calculation Complexity Comparison

To further verify the computational complexity of different point cloud registration methods, we calculated the average calculation time of successful registration of point clouds based on initial pose transformation on different datasets and compared and analysed the difference in calculation complexity, as shown in Figure 10. Because the ICP point cloud registration algorithm has poor stability with the increasing initial pose, we only compared the average calculation time of the proposed LiDAR with the IMU point cloud registration algorithm with the GICP and NDT algorithms.

**Figure 11:**
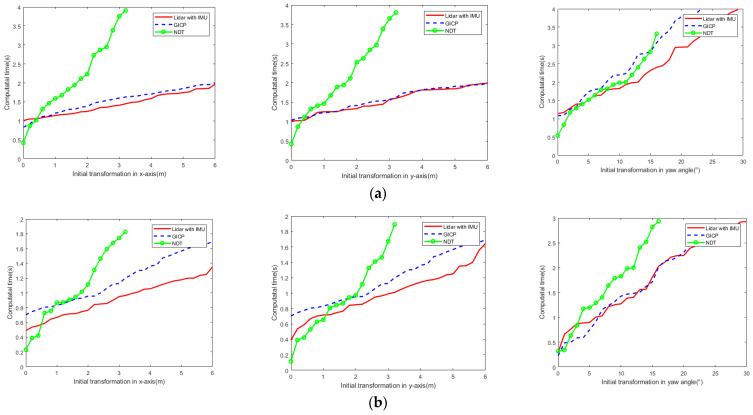

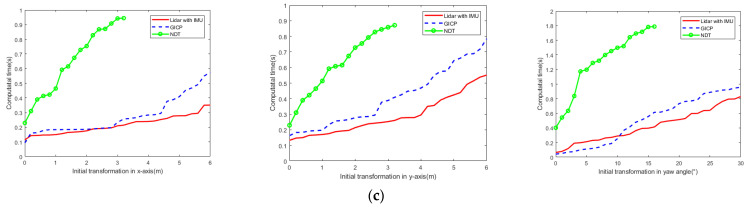
Calculation time of successful matching of different algorithms; (**left**) initial transformation in x-axis direction, (**middle**) y-axis direction, (**right**) initial yaw angle. (**a**) KITTI datasets; (**b**) Park datasets; (**c**) Coal mine roadway datasets.

Figure 11 shows that under the same conditions, the calculation time of the NDT algorithm was significantly longer than that of the other two algorithms. The calculation time of LiDAR with IMU and GICP was similar to the increase in initial transformation. However, the average calculation time of LiDAR with IMU was slower than that of GICP for smaller initial transformations, while that of LiDAR with IMU was faster than that of GICP for larger initial transformations. Notably, in the special environment of coal mine roadways, the average calculation time of point cloud registration using LiDAR with the IMU method was less than that using the GICP method.

### 4.4. Comparative Experiment of Constructing LiDAR Odometry Track Error in Coal Mine Roadway

In order to further illustrate the effect of point cloud registration performance on mobile robot laser odometers. In the coal mine roadway environment, we used our own wheeled mobile robot system equipped with VLP-16 to collect four sets of different degrees of difficulty datasets as experimental data. Using the extracted feature corner points and feature plane points, we carried out the point cloud registration experiment and focused on testing the track length error and absolute pose error of our proposed point cloud registration method integrating IMU preintegration to build the LiDAR odometry. Because LiDAR odometry constructed using ICP and NDT methods has a large drift in the coal mine tunnel environment, which has lost the significance of comparison, only GICP-based LiDAR odometry was constructed here to carry out a comparative experiment with our proposed method. The comparison of the length error and absolute position and pose error of the four groups of tracks was statistically analysed.

#### 4.4.1. Comparison of LiDAR Odometry Track Length Error

**Figure 12:**
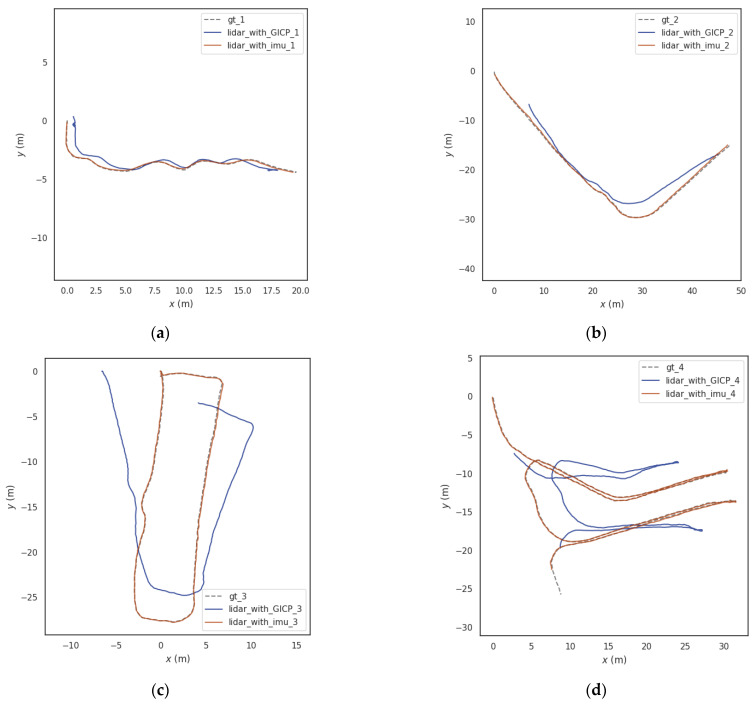
Comparison of the trajectories of LiDAR odometry constructed by different point cloud registration algorithms. (**a**) Track comparison of experiment 1; (**b**) Track comparison of experiment 2; (**c**) Track comparison of experiment 3; (**d**) Track comparison of experiment 4.

A GICP-based LiDAR odometry and a LiDAR odometry fused with IMU preintegration information were constructed on four groups of self-collected datasets and conducting a trajectory comparison experiment. By comparing and analyzing the errors of four groups of track length, the authenticity of the LiDAR odometry constructed can be reflected. In this paper, the EVO tool is used for comparative analysis. In order to further illustrate the importance of point cloud registration performance for mobile robot LiDAR odometry. The error comparison experiment of LiDAR odometry is constructed under the coal mine roadway environment. In order to ensure the fairness and rationality of the experimental comparison, the unified LOAM algorithm is used here to build the LiDAR inertial odometry, but only the front-end point cloud registration part of the LOAM algorithm is changed into the GICP registration algorithm and our registration method, and the comparative experiments are carried out on four different sets of data to verify that the LiDAR odometry constructed by our proposed point cloud registration method can better reflect the authenticity of the LiDAR odometry track, it has higher track accuracy. The comparison between the four groups of motion trajectories and true values obtained by different algorithms is shown in Figure 12. The meaning of registration error refers to the difference between the actual length of the current algorithm track and the actual value of the track. That is to say, authenticity represents the difference between the track output by the current algorithm and the actual track length and is also a quantitative analysis of the output track.

As can be seen from Figure 11, when the test scenario is relatively simple, there is little difference between the two methods. However, with the increasing difficulty of the test scenario, the two show obvious differences. When the robot moves back and forth in the coal mine roadway and the environment similarity is high, the method based on GICP is prone to point cloud registration errors. We have calculated the true length and error of the trajectory obtained by two different methods, as shown in Table 2.

According to the statistical results in Table 2, LiDAR odometery was constructed on four groups of coal mine roadway datasets to test the length error by comparing the two-point cloud registration methods, LiDAR_with_GICP and LiDAR_with_imu. The track length obtained by the LiDAR_with_imu method was closer to the true value, and the error rate was lower. Therefore, compared with the traditional point cloud registration algorithm based on GICP, the point cloud registration method based on IMU preintegration reduced the track length error by 15.33%, which can better reflect the authenticity of the LiDAR odometry track.

#### 4.4.2. Comparison of Absolute Position and Pose Error of Trajectory

In order to further improve the comparative experiment, on the basis of the above four groups of experiments, we take into account the absolute position and attitude errors obtained by rotation and translation errors, and separately count the maximum, minimum, average, root mean square error, and standard deviation five evaluation indicators. By comparing and analyzing the absolute position and pose errors of the four groups of tracks, the global consistency of the whole track can be evaluated as a whole, reflecting the track accuracy of the four groups of experiments.

The statistical results in Table 3 show that by comparing the two point cloud registration methods LiDAR_with_GICP and LiDAR_with_imu, the LiDAR odometry and the true value are constructed on four groups of coal mine roadway datasets to compare the absolute position and pose errors. Notably, the absolute error of each track counted here is the result of considering both rotation and translation under SE(3). The absolute error of the trajectory obtained by the LiDAR_with_imu method was smaller than the true value. Therefore, compared with the traditional GICP-based point cloud registration algorithm, the root mean square error (RMSE) of the trajectory was reduced by 45.04%, which proves that the IMU preintegration point cloud registration method has a higher trajectory accuracy.

#### 4.4.3. Absolute Position and Pose Error Comparison in Experiment 4

**Figure 13:**
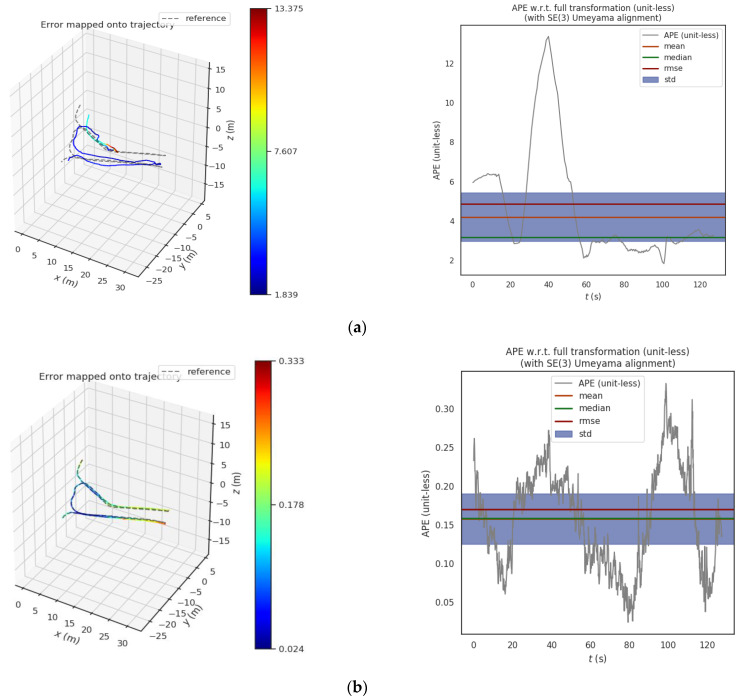
Comparison of absolute position and pose errors of different methods in Experiment 4; (**left**) the comparison between the trajectory and the true value; (**right**) the change of absolute position and pose error with time. (**a**) Comparison between method LiDAR_with_GICP track and true value; (**b**) Comparison between method LiDAR_with_imu track and true value.

In order to more clearly show the advantages of our proposed method in the special environment of coal mine roadways, only experiment four, which is more challenging and difficult, is selected here for more detailed absolute position and pose error comparison. By comparing the trajectories obtained by two different methods LiDAR_with_GICP and LiDAR_with_imu with the true values, and taking into account the changes of the absolute position and pose errors of rotation and translation errors with time, as shown in Figure 13.

**Figure 14:**
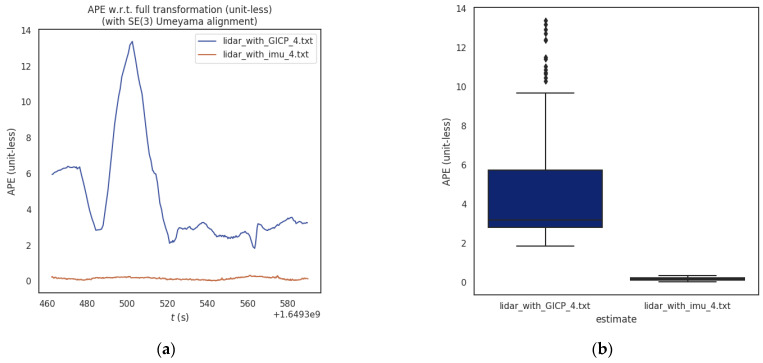
Comparative analysis of absolute position and pose error under SE(3) in Experiment 4. (**a**) Different methods consider the absolute position and pose error of translation and rotation simultaneously; (**b**) Absolute position and pose error box diagram of different methods.

In order to further test the difference between the global consistency of the trajectories obtained by the two different methods, we analyzed the absolute position and pose errors considering both rotation and translation errors under SE(3) and its corresponding box diagram are shown in Figure 14.

The statistical results show that the proposed algorithm has a smaller absolute position and pose error compared with the traditional GICP algorithm when tested with two different algorithms in the coal mine roadway experiment 4 datasets.

## 5. Conclusions

(1) A point cloud registration method with IMU preintegration was proposed. The system framework of this method mainly consists of four modules: IMU preintegration, point cloud preprocessing, point cloud frame matching and point cloud registration. In this method, the IMU preintegration error equation and the LiDAR point cloud registration error equation are constructed, and the Gauss-Newton solution was used to optimize the registration of two adjacent frames of LiDAR. The results show that our method has a higher accuracy, success rate and computational efficiency than traditional point cloud registration methods.

(2) The IMU preintegration result was introduced to correct the distortion of the original LiDAR point cloud, which solves the problem of point cloud motion distortion. The ground extraction method based on RANSAC was adopted for point cloud segmentation, which provides additional ground constraints for the z-axis displacement, and solves the problem of z-axis drift during point cloud registration; The stability of point cloud registration was improved by eliminating the unstable flaw points in the point cloud. The robustness of point cloud registration is improved by extracting feature corner points and plane points in the point cloud.

(3) In view of the special environment of coal mine roadways, the LiDAR odometry constructed by this method reduced the error of track length by 15.33% compared with the traditional point cloud registration algorithm based on GICP, which can better reflect the authenticity of the track. The root mean square error (RMSE) of the trajectory was reduced by 45.04%, which proves that this method has higher trajectory accuracy and smaller absolute position and pose error.

## Figures and Tables

**Table 1:** Absolute position and pose changes of different point cloud registration methods.

Experiment	Axis (m)	Degree (°)	LiDAR with IMU		GICP		ICP		NDT	
a	X	Roll	**0.0029**	**0.0011**	0.2811	0.0405	−1.0352	0.2291	−9.6645	0.3725
	Y	Ritch	**−0.0030**	**0.0025**	0.3343	0.0496	0.3252	0.5522	−0.3270	0.4751
	Z	Yaw	**0.0050**	**0.0094**	0.0145	−0.0556	−0.0023	−0.8689	−0.9153	−0.8260
b	X	Roll	**−0.0040**	**−0.0074**	−1.0034	0.0166	−0.9182	0.9454	−0.9926	−0.1519
	Y	Ritch	**0.0016**	**0.0022**	−0.0238	−0.0348	0.1124	−0.3685	0.1607	−0.3705
	Z	Yaw	**−0.0047**	**0.0063**	−0.0393	0.1230	−0.4297	0.1361	−0.6582	0.1308
c	X	Roll	**−0.0091**	**0.0012**	−0.0388	0.0267	−2.2227	−0.1868	−2.2593	0.5345
	Y	Ritch	**0.0037**	**0.0039**	−0.0848	−0.0236	−0.7846	−0.9348	−0.8508	−0.1745
	Z	Yaw	**0.0016**	**0.0041**	−0.1148	0.0692	−0.0483	0.7116	−0.2102	0.7126

**Table 2:** Comparison of track length error.

Number	Groundtruth	LiDAR_with_GICP		LiDAR_with_imu	
	Length (m)	Length (m)	Error (%)	length (m)	Error (%)
1	24.62	22.76	7.55	24.49	0.52
2	68.02	52.83	22.33	67.05	1.43
3	68.65	58.81	14.33	66.03	3.82
4	133.91	98.26	26.62	128.76	3.85

**Table 3:** Comparison of relative error of track.

Number	Algorithm Comparison	Ma x(m)	Min (m)	Mean (m)	RMSE (m)	Std (m)
1	LiDAR_with_GICP_1	3.138	1.879	2.481	2.495	0.291
	LiDAR_with_imu_1	2.830	1.860	2.201	2.220	0.253
2	LiDAR_with_GICP_2	9.899	2.115	4.131	4.523	1.841
	LiDAR_with_imu_2	3.039	2.084	2.680	2.692	0.248
3	LiDAR_with_GICP_3	6.946	2.629	4.497	4.684	1.310
	LiDAR_with_imu_3	1.831	0.0137	0.151	0.364	0.331
4	LiDAR_with_GICP_4	13.375	1.838	4.205	4.871	2.459
	LiDAR_with_imu_4	0.332	0.024	0.158	0.171	0.065

## Data Availability

The datasets analyzed during the current study was derived from KITTI (http://https://www.cvlibs.net/datasets/kitti/raw_data.php/) (accessed on 10 November 2022) and Park datasets (https://github.com/TixiaoShan/LIO-SAM) (accessed on 10 November 2022).
